# Close lateral internal sphincterotomy versus open lateral internal sphincterotomy for chronic anal fissure: a systematic review and meta-analysis

**DOI:** 10.1097/MS9.0000000000001593

**Published:** 2023-12-08

**Authors:** Aiman Tanveer, Sheraz Arshad, Nour Fakih, Dawood Azam Farooq, Ahmad Afyouni, Ateeba Kamran, Muhammad Imran

**Affiliations:** a University College of Medicine and Dentistry, University of Lahore, Lahore; b Karachi Medical and Dental College, Karachi, Pakistan; c Department of Natural Sciences, School of Arts and Sciences, Lebanese American University; d Faculty of Medical Sciences, Lebanese University, Hadath Campus, Beirut, Lebanon

**Keywords:** anal fissure, closed method, lateral internal sphincterotomy, meta-analysis, open method, systematic review

## Abstract

**Background:**

Lateral internal sphincterotomy (LIS) has been the gold standard for treating chronic anal fissure (CAF) that persists despite other measures. The authors aim to evaluate the effects of the close method (CLIS) of performing LIS as compared to the open method (OLIS).

**Methods:**

Databases were searched for relevant studies and results were screened to identify eligible articles, and all concerned outcomes were pooled as odd ratio (OR) or mean difference (MD) with 95% CI in the meta-analysis models using RevMan 5.4.

**Results:**

Pooled data from 16 trials with 1,711 patients with idiopathic CAF showed that the CLIS has significant lower risk of delayed fissure healing [OR: 0.28, 95% CI (0.10, 0.77), *P* = 0.01], duration of hospital stay [MD: -0.82 with 95% CI (−1.07, −0.57), *P* < 0.00001] and postoperative visual analogue pain score (VAPS) at 24 h [MD: −0.30 with 95% CI (−0.39, −0.21), *P* < 0.00001]. Also, the risk of overall complications [OR: 0.33 with 95% CI (0.19, 0.55), *P* < 0.0001], incontinence [OR: 0.28 with 95% CI (0.20, 0.38), *P* < 0.00001], and postoperative pain [OR: 0.56 with 95% CI (0.35, 0.91), *P* = 0.02] was significantly lower with CLIS.

**Conclusion:**

CLIS is a safer option than OLIS for treating anal fissure. The risk of delayed fissure healing, incontinence, post-op pain and overall complication was significantly lower. However, the risk of surgical site infection, postoperative bleeding and recurrence did not differ. Future research with more prolonged follow-up is necessary to document recurrence reliably.

## Introduction

Highlights

- This meta-analysis compares the effectiveness and complications of open (OLIS) and closed (CLIS) lateral internal sphincterotomy (LIS) for chronic anal fissures (CAF), seeking alternatives to the gold standard treatment for faster healing with lower risk of incontinence.
- CLIS is more effective and safer than OLIS for CAF treatment.
- CLIS is associated with lower risk of delayed fissure healing, shorter hospital stays, and improved postoperative VAPS.
- Closed procedure reduces overall complications and incontinence risk compared to open approach.
- No significant differences in bleeding, surgical site infection, and recurrence between CLIS and OLIS
- Future research with prolonged follow-up is necessary to document recurrent reliably.

An anal fissure is defined as a longitudinal split in the distal anoderm, spanning from the anal verge to the dentate line. It manifests with intense and excruciating pain during bowel movements and occasional rectal bleeding, typically appearing as a streak of blood^1^. This condition occurs when the delicate anal mucosa is disrupted, primarily due to trauma resulting from factors like the passage of hard stools or excessive straining during defecation. Consequently, pain ensues, accompanied by spasms of the anal sphincter, leading to a compromised blood supply to the affected area and hindering the healing process^2^. Primary fissures, predominantly found at the posterior midline (about 90% of cases) or occasionally at the anterior midline mostly in females, often lack an identifiable cause. On the other hand, secondary fissures arise from various systemic conditions such as inflammatory bowel disease, malignancies, HIV infection, or tuberculosis, and they require addressing the underlying cause for proper healing^3,4^. If left untreated, anal fissures can lead to complications such as secondary infections, abscess formation, anal stenosis, hypertrophied papilla, fistulas development and can progress to a chronic state where persistent ischaemia results in fibrosis at the edges of the fissure, thereby impeding the effective healing^4^.

The American Society of Colon and Rectal Surgeons recommends conservative management, including the use of stool softeners, a high-fibre diet, sitz baths, and topical analgesia with steroids, as the initial approach for treating acute anal fissure^5^. However, conservative management is often ineffective for chronic anal fissures (CAF), necessitating further treatment options. Lateral internal sphincterotomy (LIS) has emerged as the gold standard treatment for CAF, demonstrating superior outcomes in terms of symptom relief, early recovery and low incidence of recurrence when performed by proctology trained surgeons, compared to medical management approaches such as calcium channel blockers, trinitroglycerin, and botulinum toxin injections^6–8^. Despite its effectiveness, LIS carries potential downsides, with anal incontinence being the most concerning complication^6,9^. Advances in LIS techniques, including both open and closed approaches, have been developed to mitigate these adverse outcomes.

Previous systematic reviews have shown comparable efficacy between closed LIS (CLIS) and open LIS (OLIS) in terms of healing rates^10,11^, but studies comparing other efficacy and complication outcomes have yielded mixed results. Therefore, this meta-analysis focuses exclusively on comparing CLIS and OLIS regarding their efficacy and complication outcomes in CAF patients by conducting a comprehensive analysis of the available evidence till so far.

## Methodology

### Protocol registration

The protocol for this review has been registered and published in PROSPERO. Our systematic review and meta-analysis adhered to the guidelines provided by the PRISMA statement^12^ and the Cochrane Handbook for Systematic Reviews and Meta-Analyses^13^, Supplemental Digital Content 1, http://links.lww.com/MS9/A321 and AMSTAR 2 (assessing the methodological quality of systematic reviews, Supplemental Digital Content 1, http://links.lww.com/MS9/A321 ^14^. PRISMA checklist is illustrated in Supplementary table 1, Supplemental Digital Content 1, http://links.lww.com/MS9/A321.

### Data sources and search strategy

We performed a thorough electronic search for relevant literature by utilizing databases, including PubMed (MEDLINE), the Cochrane Central Register of Controlled Trials (CENTRAL) from the inception of these databases to 1 June 2023. Complete search strategy has been provided in Supplementary table 2, Supplemental Digital Content 1, http://links.lww.com/MS9/A321. We did not use any limitations on the search. Citation searching of the included articles was also done.

### Eligibility criteria

A PICO criterion was used to include randomized and non-randomized clinical trials: population (P): patients of any age, gender having an idiopathic chronic anal fissure (>6 weeks) whether it is located posterior, anterior or any other location of anal canal without any associated anal stenosis, abscess, fistula, haemorrhoids, inflammatory bowel disease and malignancy; intervention (I): CLIS; comparator (C): OLIS; outcomes (O): efficacy outcomes including the risk of delayed healing, postoperative visual analogue pain score, duration of hospital stay, duration of intervention, and postoperative complication outcomes including the risk of incontinence, pain, bleeding, infection and recurrence.

We excluded the randomized and non-randomized clinical trials involving patients with anal fissure having associated inflammatory bowel disease, malignancy, abscess, fistula or haemorrhoids. Observational comparative studies were also excluded.

### Study selection

Search results from all the databases were imported to Covidence.org and duplicates were removed automatically. The remaining records were screened independently by two authors (S.A., A.T.) and any conflict between them was resolved by a third author (D.A.F.). The screening was done in two steps: (i) title and abstract screening to determine the relevance of the study for this meta-analysis, and (ii) full-text screening according to the inclusion criteria for the final eligibility for qualitative and quantitative analysis.

### Data extraction

Data were collected independently by four review authors (S.A., A.T., D.A.F., and A.K.) and extracted into a uniform data extraction Excel sheet. The extracted data included characteristics of the included studies like duration of study, country of origin, total number of participants, follow-up period and inclusion criteria and participants baseline characteristics like mean age, sex, location of fissure and number of participants across intervention and comparator group and concerned outcome measures. Any disagreement between the review authors was resolved by consensus or consultation.

### Risk of bias and quality assessment

Four reviewers (S.A., A.T., D.A.F., and A.K.) independently assessed the risk of bias in the included trials using Joanna Briggs Institute (JBI) critical appraisal tool for randomized controlled trials and Quasi-experimental studies. Each question was answered as yes, no, unclear, or not applicable while an author’s judgment of risk of bias for a study was to include, exclude or seek further information based on answers to the questions. Any disagreement between the review authors was resolved by consensus or consultation.

### Statistical analysis

The RevMan v5.4 software was used for statistical analysis. To combine the outcomes for dichotomous outcomes, the odd ratio (OR) was used while the mean difference (MD) was used for continuous outcomes. Both were calculated with a 95% CI using the fixed-effects model. However, the random-effects model was used in case of significant heterogeneity. The presence and extent of heterogeneity were evaluated using the χ^2^ and I^2^ tests, respectively. Heterogeneity was considered significant if the alpha level for the χ^2^ test was below 0.1, while the I^2^ test results were interpreted as follows: not significant for 0–40%, moderate heterogeneity for 30–60%, and substantial heterogeneity for 50–90%. In case of substantial heterogeneity, a leave-out one sensitivity analysis was conducted. A *p* value less than 0.05 was considered statistically significant.

## Results

### Search results and study selection

**Figure 1:**
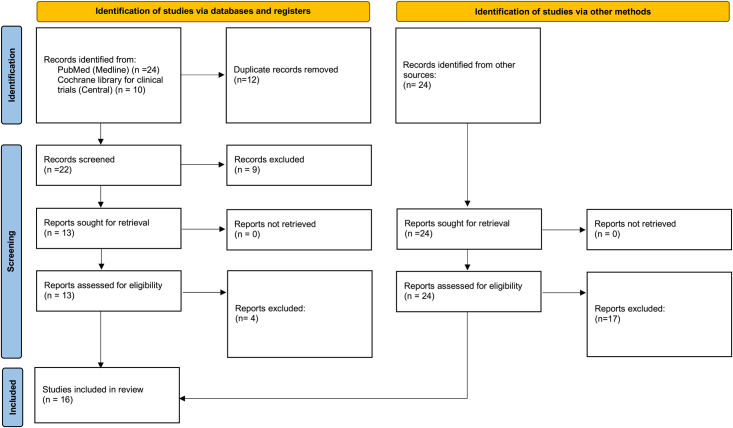
PRISMA flow chart of the screening process.

Search results and studies selection process is shown in Fig. 1. As a result of the screening process, 16 studies^15–31^ met the inclusion criteria for this meta-analysis.

### Characteristics of included studies and participants:

Among sixteen included studies (*n*=16), only one study was quasi experimental while all others were primarily prospective randomized clinical trials with a total of 1711 participants. Among them, 856 (50.02%) underwent CLIS and 855 (49.98%) OLIS. Overall, male with posterior midline anal fissures were predominant. Characteristics of the included studies and the participants’ baseline characteristics are shown in Tables 1 and 2 respectively.

**Table 1:** Characteristics of the included studies

Study ID	Country	Study design	Duration of study	Total number of participants (*N*)	Inclusion criteria	Follow-up period	Outcomes
Seleem *et al*. 2022^27^	Egypt	Prospective randomized study	June 2017–May 2019	150	Patients above age 14 years or 60 years old, patients without previous rectal surgery, and associated Inflammatory bowel disorders or malignancies.	1 Year	Pain intensity, post-op complication and fissure healing
Shah *et al*.^28^	Pakistan	Clinical randomized comparative study	Feb 2019–Feb 2020	168	NR	NR	Faecal incontinence and healing rate
Abdul Nasir *et al*. 2022^25^	Afghanistan	Prospective randomized study	Dec 2019–Dec 2020	80	Patients of both sexes between ages of 25–60 years old who presented with CAF	8 months	Post-op complications
Jabran *et al*. 2021^30^	Pakistan	Quasi-experimental study	June 2017–May 2018	100	Irrespective of age and gender diagnosed with chronic anal fissure	6 weeks	Post-op complications, hospital stay
Motto *et al*.^24^	Cameroon	Prospective Randomized comparative study	15 months duration	63	Adult patients with chronic anal fissure without other associated anorectal pathologies	6 months	Post-op complications,
Hasnat *et al*. 2018^31^	Bangladesh	Clinical randomized comparative study	April 2015–Sep2015	80	Age more than 18 years without previous anorectal disorders	6 weeks	Post-op pain and healing
FIDA *et al*. 2017^15^	Pakistan	Randomized clinical study	Jan 2016–July 2017	100	Patients with age 20 years to 70 years without any other anorectal pathologies	No follow-up	Post-op. pain , faecal incontinence and hospital stay
Anandaravi *et al*.^18^	India	Prospective Randomized study	Jan 2015–Jun 2016	100	Patients with chronic anal fissure with age between 20 and 50 regardless of the gender	6 months	Post-op pain, bleeding, infection, incontinence and recurrence
Ghayas *et al*.^21^	Pakistan	Randomized control trail	Feb 2011–Aug2011	94	Age above 12 years with either gender with chronic anal fissure	5th post-op day	Faecal incontinence
Al Sanabani *et al*.^16^	Yemen	Prospective randomized study	Jan 2007–Dec 2010	205	Patients diagnosed with CAF irrespective of age , gender and without any other anorectal pathologies	6 months	Post-op complications
Gupta *et al*.^22^	India	Prospective Randomized Study	Oct 2010–Aug 2012	136	All. patients of both sexed between ages of 15–70 years presented with CAF	1 year	Post-op complications, hospital stay
Akeel *et al*. 2010^17^	Iraq	Prospective randomized study	Jan 2006–Oct 2008	100	Patients diagnosed with CAF ,age between 20 and 50 years in both sexes	NR	Pain, bleeding, infection, incontinence, recurrence
Wiley *et al*.^29^	Australia	Prospective randomized study	Jan 1997–Feb 2001	79	All patients with CAF in whom conservative medical treatment had failed and who were suitable for Lateral internal sphincterotomy	6 Weeks	Healing rate, pain score,continenece and complications
Arroyo *et al*.^19^	Spain	Prospective randomized study	Jan 1998–Jan 2000	80	All patients diagnosed with chronic anal fissure	2 years	Healing rate, post-op complications, manometric pressure
Kortbeek *et al*. 1988^23^	Canada	Randomized comparative study	July 1988–Dec1989	112	All patients diagnosed with chronic anal fissure	6 weeks	Length of hospital stay, complications, fissure healing, and continence
Sanniyasi *et al*.^26^	India	Prospective randomized study	2013–2014	64	Patients diagnosed with CAF irrespective of age , sex and without any other anorectal pathologies	6 months	Recurrence, post-op complications

CAF, chronic anal fissure; NR,not reported; Post-op, postoperative.

**Table 2:** Baseline characteristics of the participants

	No. Participants, *N*		Sex, *N* (%)				Age (years), mean (SD)		Location of fissure, *N* (%)					
			CLIS		OLIS				CLIS			OLIS		
Study ID	CLIS	OLIS	Male	Female	Male	Female	CLIS	OLIS	Posterior	Anterior	other	Posterior	Anterior	Other
Seleem *et al*.^27^	80	70	38 (55.9)	30 (44.1)	43 (63.2)	25 (36.8)	40.88 ± 11.80	39.38 ± 12.96	66 (82.5)	11 (13.75)	3 (3.75)	67 (95.7)	2 (2.9)	1 (1.42)
Shah *et al*.^28^	84	84	67(80)	17 (21)	65(77)	19(23)	33	36	NR	NR	NR	NR	NR	NR
Abdul Nasir *et al*. 2022^25^	40	40	M=61		F=19		Mean age= 42 years.		P=71		A=8		O=1	
Zafar *et al*.^30^	50	50	17 (34)	33 (66)	15 (30)	35 (70)	28.68±7.64	33.57±7.2	NR	NR	NR	NR	NR	NR
Motto *et al*.^24^	31	32	17 (26.9)	14 (22.22)	17 (26.9)	15 (23.4)	35.36±10.16	35.36±10.16	49 (77.77)	9 (14.28)	2 (3.17)	49 (77.77)	9 (14.28)	2 (3.17)
Hasnat *et al*. 2018^31^	40	40	14 (35.0)	26 (65.0)	13 (32.5)	27 (67.5)	34.73±8.88	38.18±12.05	33 (82.5.0)	6 (15.0)	1 (2.5)	34 (85.0)	4 (10.0)	2 (5.0)
FIDA *et al*. 2017^15^	50	50	32 (64.0)	18 (36.0)	35 (70)	15 (30)	39.12±12.34	41.50±11.6	47 (94.0)	2 (4.0)	1 (2.0)	46 (92)	2 (4)	2 (4)
Anandaravi *et al*.^a^ ^18^	50	50	M=68		F=32		NR		P=90		A=10		O=0	
Ghayas *et al*.^21^	47	47	40 (85.1)	7 (14.9)	81 (86.2)	6 (12.8%)	38.83±14.56	38.83±14.56	NR	NR	NR	NR	NR	NR
Al Sanabani *et al*.^16^	100	105	0	100	0	105	32.4±8.19	31.9±6.94	NR	NR	NR	NR	NR	NR
Gupta *et al*.^22^	68	68	38 (55.9)	30 (44.1)	43 (63.2)	25 (36.8)	42.2	42.2	56 (82.4)	9 (13.2)	3 (4.4)	65 (95.6)	2 (2.9)	1 (1.5)
Akeel *et al*. 2010^a^ ^17^	50	50	M=76 (76%)		F=24 (24%)		Mean Age= 35		P=89		A=10		O=1	
Wiley *et al*.^29^	38	41	16	22	20	21	45.1 (±16.3)	47.9(±16)	NR	NR	NR	NR	NR	NR
Arroyo *et al*.^19^	40	40	26 (65)	14 (35)	29 (72.5)	11 (27.5)	40 +- 15	38 +-14	31 (77.5)	9 (22.5)	0	28 (70)	12 (30)	0
Kortbeek *et al*. 1988^23^	58	54	24	34	24	30	36.6	40.8	40	15	3	41	9	4
Sanniyasi *et al*.^a^ ^26^	30	34	M=41		F=23		Mean Age= 34 ± 5 years.		P=60%		A=36%		O=4%	

A, anterior; CLIS, closed Lateral internal sphincterotomy; F, female; M, male; *N* (%), number (percentage); NR, not reported; O, others; OLIS, open lateral internal sphincterotomy; P, posterior.

a Studies with overall baseline data of participants instead of group distribution.

### Risk of bias

Risk of Bias among the included studies has been shown in Table 3. Due to the nature of intervention, blinding of the participants and personnel was not methodologically feasible in the included studies and hence, showed some risk of bias. However, true randomization and comparable baseline characteristics between the intervention and comparator group was achieved in the majority of the included studies indicating no risk of bias in these domains.

**Table 3:** Risk of bias assessment using Joanna Briggs Institute (JBI) critical appraisal tool for randomized controlled trials

	Study ID														
Domains for ROB assessments	Seleem *et al.* ^27^	Shah et al 2022^28^	Abdul Nasir *et al.* 2022	Georges Bwelle Motto *et al.* 2021	Hasnat *et al.* 2018	FIDA *et al.* 2017	Anandaravi *et al.* ^18^	Nighat Ghayas *et al.* 2015	Al Sanabani *et al.* ^16^	Gupta *et al.* ^22^	Akeel *et al.* 2010	Wiley *et al.* ^29^	Arroyo *et al.* ^19^	Kortbeek *et al.* 1988	Sanniyasi *et al.* ^26^
1. Was true randomization used for assignment of participants to treatment groups?	Unclear	Yes	Unclear	Yes	Yes	Yes	Yes	Yes	Yes	Yes	Yes	Yes	Yes	Yes	Yes
2. Was allocation to treatment groups concealed?	Unclear	Unclear	Yes	Unclear	Unclear	Unclear	Unclear	Yes	Unclear	Yes	Yes	Yes	Unclear	Unclear	Yes
3. Were treatment groups similar at the baseline?	Yes	Yes	unclear	Unclear	yes	Yes	Yes	Yes	Yes	Yes	Yes	Yes	Yes	Yes	yes
4. Were participants blind to treatment assignment?	Unclear	No	Yes	Unclear	Yes	Unclear	Unclear	Unclear	Unclear	Yes	Yes	Yes	Unclear	Unclear	Yes
5. Were those delivering treatment blind to treatment assignment?	No	No	No	No	No	No	No	No	No	No	No	No	No	No	No
6. Were outcomes assessors blind to treatment assignment?	No	No	No	No	No	No	No	No	No	No	No	No	No	No	No
7. Were treatment groups treated identically other than the intervention of interest?	Yes	Yes	Yes	Yes	Yes	Yes	Yes	Yes	Yes	Yes	Yes	Yes	Yes	Yes	Yes
8. Was follow-up complete and if not, were differences between groups in terms of their follow-up adequately described and analyzed?	Yes	Yes		Yes	Yes	Yes	Yes	Yes	Yes	Yes	Yes	No	Yes	Yes	Yes
9. Were participants analyzed in the groups to which they were randomized?	N/A	N/A	N/A	N/A	N/A	N/A	N/A	N/A	N/A	N/A	N/A	N/A	N/A	N/A	N/A
10. Were outcomes measured in the same way for treatment groups?	Yes	Yes	Yes	Yes	Yes	Yes	Yes	Yes	Yes	Yes	Yes	Yes	Yes	Yes	Yes
11. Were outcomes measured in a reliable way?	Yes	Yes	Yes	Yes	Yes	Yes	Yes	Yes	Yes	Yes	Yes	Yes	Yes	Yes	Yes
12. Was appropriate statistical analysis used?	Yes	Yes	Yes	Yes	Yes	Yes	Yes	Yes	Yes	Yes	Yes	Yes	Yes	Yes	Yes
13. Was the trial design appropriate, and any deviations from the standard RCT design (individual randomization, parallel groups) accounted for in the conduct and analysis of the trial?	Yes	Yes	yes	Yes	Yes	Yes	Yes	Yes	Yes	Yes	Yes	Yes	Yes	Yes	Yes
Overall Appraisal:	Included	Included	Included	Included	Included	Included	Included	Included	Included	Included	Included	Included	Included	Included	Included

Each domain answered as “Yes”, “No” , “unclear” or “ Not applicable” while overall appraisal of the risk of bias for a study marked as “include”, “exclude” or “seek further information” based on answers to the questions. N/A; Not applicable.

No” , “unclear” or “ Not applicable” while overall appraisal of the risk of bias for a study marked as “include”, “exclude” or “seek further information” based on answers to the questions. N/A; Not applicable.

NA, not applicable.

### Outcomes

#### Efficacy outcomes

**Figure 2:**
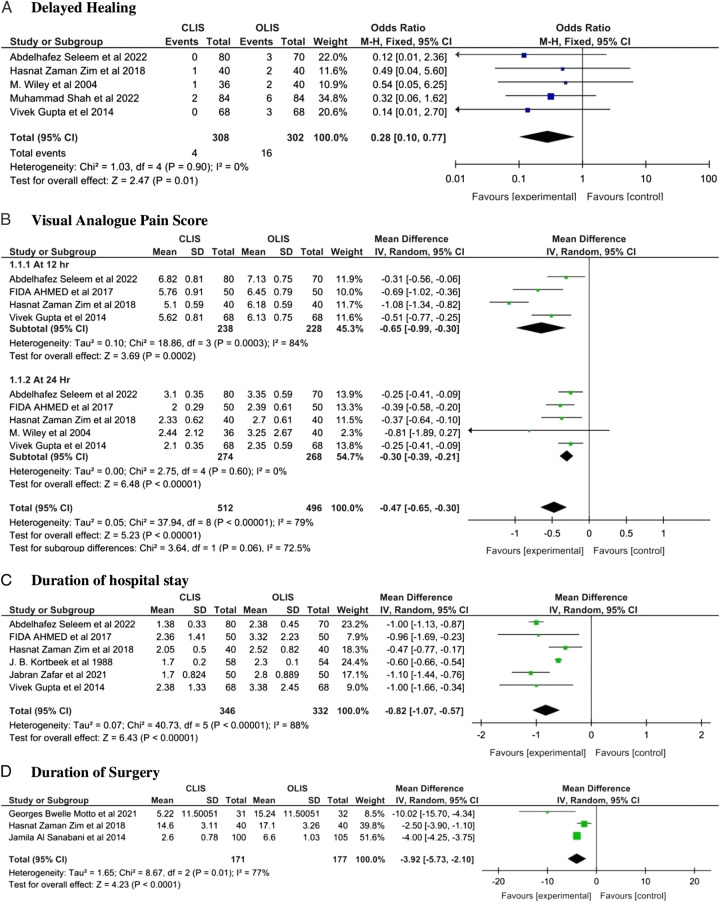
Efficacy outcomes forest plot (A) delayed healing (B) Visual Analogue Pain score (C) duration of hospital stay (D) duration of surgery. CLIS, closed Lateral internal sphincterotomy, OLIS, open lateral internal sphincterotomy.

Our meta-analysis showed a significant reduction in the risk of delayed fissure healing [OR: 0.28 with 95% CI (0.10, 0.77), *P* = 0.01] with CLIS as compared to OLIS. Also, the duration of hospital stay [MD: −0.82 with 95% CI (−1.07, −0.57), *P* < 0.00001], postoperative visual analogue pain score (VAPS) at 12 h [MD: −0.65 with 95% CI (−0.99, −0.30), *P* = 0.0002], at 24 h [MD: −0.30 with 95% CI (−0.39, −0.21), *P* < 0.00001], and duration of surgery [MD: −3.92 with 95% CI (−5.73, −2.10), *P* < 0.0001] was significantly lower with CLIS as compared to OLIS as shown in Fig. 2.

Our results were homogenous for delayed healing (*P* = 0.90; I² = 0%), and visual analogue pain score at 24 h (*P* = 0.60; I² = 0%), but heterogeneous visual analogue pain score at 12 h (*P* = 0.0003; I² = 84%), duration of hospital stay (*P* < 0.00001; I² = 88%) and duration of surgery (*P* = 0.01; I² = 77%). However, the heterogeneity was not resolved for the duration of hospital stay by sensitivity analysis (Table supplementary 3, Supplemental Digital Content 1, http://links.lww.com/MS9/A321).

#### Safety outcomes

**Figure 3:**
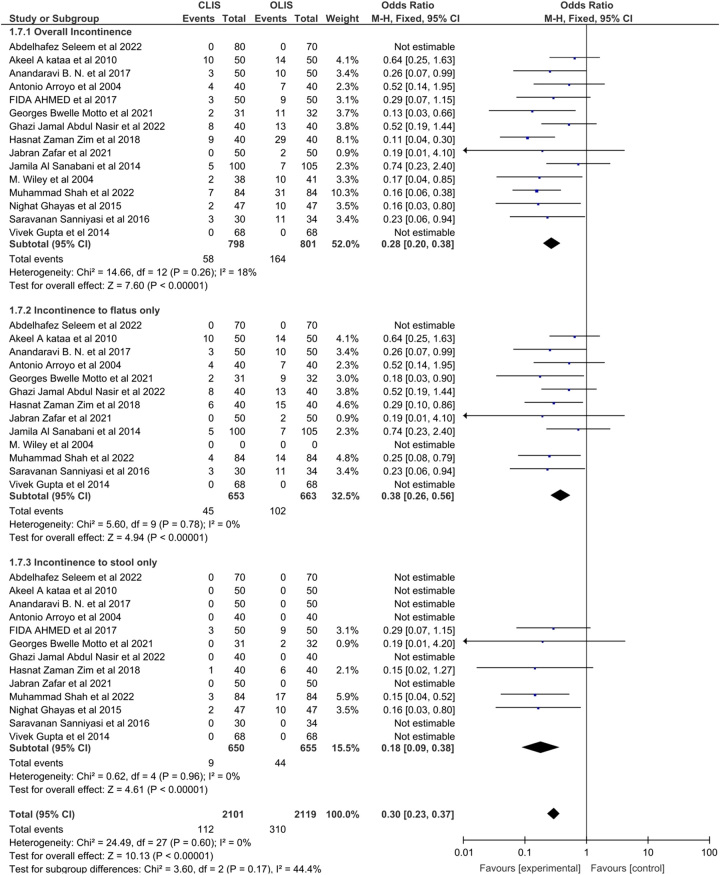
Incontinence forest plot. CLIS, closed Lateral internal sphincterotomy; OLIS, open lateral internal sphincterotomy.

**Figure 4:**
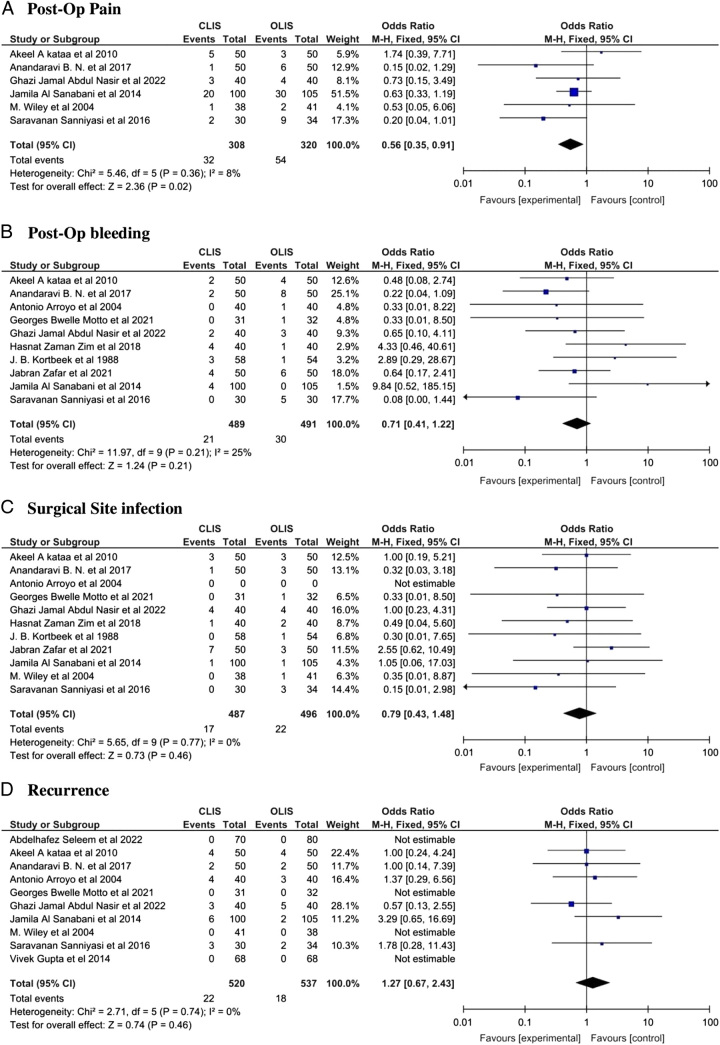
Complication outcomes forest plot (A) postoperative pain (B) postoperative bleeding (C) surgical site infection (D) recurrence. CLIS, closed Lateral internal sphincterotomy; OLIS, open lateral internal sphincterotomy.

Our meta-analysis showed significant lower risk of overall complications [OR: 0.33 with 95% CI (0.19, 0.55), *P* < 0.0001] with CLIS as compared to OLIS (Figure Supplementary 1, Supplemental Digital Content 1, http://links.lww.com/MS9/A321). Incontinence was the major complication noted and overall incontinence [(OR: 0.28 with 95% CI (0.20, 0.38), *P* < 0.00001], incontinence to flatus only [OR: 0.38 with 95% CI (0.26, 0.56), *P* < 0.00001] and incontinence to stool only [OR: 0.18 with 95% CI (0.09, 0.38), *P* < 0.00001] was significantly lower with CLIS as shown in Fig. 3. Postoperative pain [OR: 0.56 with 95% CI [(0.35, 0.91), *P* = 0.02] was also significantly lower in the CLIS group. However, postoperative bleeding [OR: 0.71 with 95% CI (0.41, 1.22), *P* = 0.21], infection [OR: 0.79 with 95% CI (0.43, 1.48), *P* = 0.46], and recurrence [OR: 1.27 with 95% CI (0.67, 2.43), *P* = 0.46] did not differ between the two groups as shown in Fig. 4.

Our results were homogenous for overall incontinence (*P* = 0.26; I² = 18%), incontinence to flatus only (*P* = 0.78; I² = 0%), incontinence to stool only (*P* = 0.96; I² = 0%), postoperative pain (*P* = 0.36; I² = 8%), recurrence (*P* = 0.74; I² = 0%), infection (*P* = 0.77; I² = 0%) and postoperative bleeding (*P* = 0.21); I² = 25%). However, results for overall post-op complications were heterogeneous (*P* < 0.00001; I² = 73%) and the heterogeneity was not resolved by sensitivity analysis (Table Supplementary 4, Supplemental Digital Content 1, http://links.lww.com/MS9/A321).

## Discussion

Anal fissure is a prevalent condition that affects a considerable number of individuals, with an estimated 342 000 new cases reported in the United States each year^32^. CAF can cause debilitating symptoms, including severe anorectal pain, sharp discomfort during bowel movements, and persistent post-defecation pain^33^. It is noteworthy that anal fissures can occur in people of all age groups, with equal incidence observed between males and females^34^. Regarding the management of anal fissures, various treatment options are available depending on the severity of symptoms and the response to initial interventions. Conservative measures, such as dietary modifications, fibre supplements and medicinal treatments, such as topical nitroglycerin or calcium channel blockers, botulinum toxin injections may be prescribed to relax the anal sphincter muscle, improve blood flow aimed to promote healing, alleviate symptoms, and facilitate stool passage^35^. In cases where conservative and medicinal approaches fail to achieve satisfactory outcomes, surgical interventions such as lateral internal sphincterotomy is considered^36^.

A recent network meta-analysis of 69 randomized controlled trials highlights LIS as the most effective treatment option for anal fissure, consistently demonstrating the highest odds of healing compared to botulinum toxin and medical therapy at all follow-up time points^6^. It is important to note that LIS, although highly effective in promoting healing, was associated with a higher risk of faecal and flatus incontinence^6,9^. To overcome these complications, advancements in basic LIS have been made such as the open LIS (OLIS) and closed LIS (CLIS). Two systematic reviews and meta-analysis conducted previously found both methods to be equally efficacious in terms of healing rate^10,11^. However, the trails comparing these methods present mixed results in terms of other efficacy and complication outcomes. Therefore, the objective of systematic review was to exclusively assess efficacy and complication outcomes between CLIS and OLIS.

The results of the meta-analysis provide compelling evidence supporting the superiority of CLIS over OLIS as the preferred surgical approach for the treatment of CAF. The analysis revealed several key findings, shedding light on the efficacy and safety advantages associated with CLIS. In terms of efficacy outcomes, CLIS demonstrated a significantly higher likelihood of promoting faster healing of anal fissures compared to OLIS suggesting that the closed approach may expedite the resolution of CAF, leading to quicker relief of symptoms and improved patient outcomes. Moreover, patients who underwent CLIS exhibited several favourable factors contributing to enhanced efficacy. These included shorter hospital stays, reduced postoperative pain levels at both 12 and 24 h, and a shorter duration of surgery. These collective benefits highlight the superior postoperative recovery and diminished surgical burden associated with CLIS. Regarding safety outcomes, CLIS demonstrated a notable advantage in terms of overall complications when compared to OLIS. Notably, the risk of incontinence, encompassing both flatus and stool, was significantly lower in the CLIS group. This finding underscores the potential of CLIS to preserve sphincter function and minimize the risk of postoperative incontinence. Additionally, postoperative pain was significantly lower in the CLIS group, indicating better pain management and potential improvements in patient satisfaction. However, it is important to note that certain safety outcomes, including postoperative bleeding, infection, and recurrence rates, did not exhibit significant differences between CLIS and OLIS.

We found two systematic reviews and meta-analyses studying the most efficacious treatment for CAF published in 2017 and 2011^10,11^. Both studies compared CLIS with OLIS but didn’t include all the clinical trials presented in our paper. Interestingly, both systematic reviews found that open and closed LIS are equally effective and the combined analyses of open versus closed partial lateral internal sphincterotomy show little difference between the two procedures in both the fissure persistence and the risk of incontinence. Interestingly, these statements contradict our findings which could be attributed to the smaller number of RCTs included in these meta-analyses.

The strengths of our study are numerous. To our knowledge, our paper is the first systematic review and meta-analysis comparing the efficacy and safety of CLIS and OLIS exclusively, providing brand new findings of a high confidence level. We maintained the strict inclusion and exclusion criteria, and a rigorous selection process of high-quality studies with a low risk of bias. Despite these strengths, it is important to address certain limitations. One limitation of our study is the heterogeneity observed in some of the analyzed outcomes such as visual analogue pain score at 12 h, duration of surgery, duration of hospital stay, and overall postoperative complications. It should be noted that the duration of hospital stay and the overall post-op complications were not resolved by sensitivity analysis. The heterogeneity observed may stem from variations in study design, patient characteristics, variability in surgical expertise, time and location factors of the studies included. Moreover, we did not follow a specific criteria to include studies based on follow-up period. The included studies have variable follow-up duration ranging from six weeks to 2 years, hence our findings regarding the recurrence should be interpreted cautiously as fissure may take a long time for recurrence. Also, none of the included study has separately mentioned the outcomes of the anterior and posterior fissures, however true randomization was achieved in all the included studies between the two groups in this regard and overall pooled population was majority of the male with posterior midline anal fissures. Moreover, we only used two databases to conduct our search. Additionally, we included only patients with idiopathic CAF in our review and excluded patients with CAF associated with anal stenosis, abscess, fistula, haemorrhoids. As a result, the efficacy and safety of this intervention in patients with these associations of CAF are unknown. Further research is needed to compare the efficacy and safety of different LIS approaches in CAF associated with the conditions mentioned above.

Our study provides clinicians and surgeons with valuable evidence-based guidance for decision-making in the management of CAF by emphasizing the efficacy and safety benefits of CLIS over OLIS. This information can help in selecting the best surgical technique, optimizing patient outcomes, and improving the overall quality of care for CAF patients. Our findings have implications that go beyond the specific surgical technique. They add to the existing body of knowledge on anal fissure management and have the potential to influence clinical guidelines and practice patterns. Adopting CLIS as the preferred approach could result in better patient outcomes, lower healthcare costs, and higher patient satisfaction. However, further studies with more prolonged follow-up are necessary to document recurrence reliably.

## Conclusion

Our systematic review and meta-analysis indicate that CLIS is more effective and safer than OLIS. CLIS has a lower risk of delayed fissure healing, duration of hospital stay and better postoperative VAPS. Furthermore, there was less risk of overall complications and incontinence with the closed procedure. Moreover, bleeding, infections, and recurrence complications showed no difference between the two approaches. These findings have implications for clinical practice and can optimize the choice of surgical treatment in CAF management. However, it is important to consider that our analysis focused specifically on patients with idiopathic CAF, excluding patients with CAF associated with anal stenosis, abscess, fistula, haemorrhoids, inflammatory bowel disease, and malignancy. Further investigation needs to be done in patients with the associations mentioned before. This will contribute to a deeper understanding of the optimal management of CAF of a wider population in an evidence-based manner.

## Ethics approval and consent to participate

Not applicable.

## Consent for publication

Not applicable.

## Source of funding

We received no funding for this study.

## Author contributions

A.T. and S.A. conceived the idea and M.I. designed the research workflow. A.T., S.A., D.A.F., A.K. searched the databases and screened the retrieved records, extracted relevant data, assessed the risk of bias. M.I. performed the analysis. N.F., A.F., A.T., S.A., and D.A.F. wrote the final manuscript. M.I. supervised the project. All authors have read and agreed to the final version of the manuscript.

## Conflicts of interest disclosure

The author declared no conflicts of interest.

## Research registration unique identifying number (UIN)

CRD42023441640.

## Guarantor

Nour Fakih , Mohammad Imran.

## Availability of data and materials

Not applicable.

## Provenance and peer review

Not commissioned, externally peer-reviewed

## Supplementary Material
